# Early Dyskinesias in Parkinson’s Disease Patients With Parkin Mutation: A Primary Corticostriatal Synaptopathy?

**DOI:** 10.3389/fnins.2019.00273

**Published:** 2019-03-26

**Authors:** Jenny Sassone, Flavia Valtorta, Andrea Ciammola

**Affiliations:** ^1^Division of Neuroscience, IRCCS San Raffaele Scientific Institute, Milan, Italy; ^2^Vita-Salute San Raffaele University, Milan, Italy; ^3^Department of Neurology, IRCCS Istituto Auxologico Italiano, Milan, Italy

**Keywords:** synaptopathy, glutamatergic transmission, parkin, dyskinesia, striatum

## Abstract

Mutations in the *PARKIN* gene cause early-onset Parkinson’s disease (PD). Despite the high proportion of still missing phenotyping data in the literature devoted to early-onset PD, studies suggest that, as compared with late-onset PD, *PARKIN* patients show dystonia at onset and extremely dose-sensitive levodopa-induced dyskinesia (LID). What pathophysiological mechanisms underpin such early and atypical dyskinesia in patients with *PARKIN* mutations? Though the precise mechanisms underlying dystonia and LID are still unclear, evidence suggests that hyperkinetic disorders in PD are a behavioral expression of maladaptive functional and morphological changes at corticostriatal synapses induced by long-term dopamine (DA) depletion. However, since the dyskinesia in *PARKIN* patients can also be present at onset, other mechanisms beside the well-established DA depletion may play a role in the development of dyskinesia in these patients. Because cortical and striatal neurons express parkin protein, and parkin modulates the function of ionotropic glutamatergic receptors (iGluRs), an intriguing explanation may rest on the potential role of parkin in directly controlling the glutamatergic corticostriatal synapse transmission. We discuss the novel theory that loss of parkin function can dysregulate transmission at the corticostriatal synapses where they cause early maladaptive changes that co-occur with the changes stemming from DA loss. This hypothesis suggests an early striatal synaptopathy; it could lay the groundwork for pharmacological treatment of dyskinesias and LID in patients with *PARKIN* mutations.

## Introduction

Loss of function mutations in the *PARKIN* gene cause autosomal recessive juvenile parkinsonism (ARJP) (Kitada et al., 1998). Autopsy studies have shown that neuropathological changes in patients with *PARKIN* mutations differ from those observed in idiopathic Parkinson’s disease (iPD). Unlike iPD, neuronal loss is greater in the substantia nigra pars compacta (SNpc) than in the locus coeruleus in most parkin cases. Also, Lewy bodies are not detected in the majority of parkin cases, suggesting a difference in the pathogenic processes between *PARKIN*-related disease and iPD (Schneider and Alcalay, 2017). Also, *PARKIN*-associated, early-onset PD is phenotypically distinct from iPD. Recent reviews in the clinical literature on genetic PD have pointed to the lack of a detailed description of individual phenotypic features (Kasten et al., 2017, 2018). Evidence suggests, however, that unlike late-onset PD patients, parkin patients display dystonia at early disease stages and dyskinesia at exceedingly low dosages of levodopa (Wickremaratchi et al., 2011). Early dystonia and dyskinesia are also reported in other early-onset genetic forms of PD such as *PINK1* and *DJ1* (Lohmann et al., 2009). Since *PARKIN* mutations account for about three-quarters of PD cases with an age at onset of 30 years or younger, understanding the pathophysiological mechanisms that underlie early and atypical dystonia and dyskinesia in *PARKIN* patients is a key step toward achieving a more personalized treatment for this genetic condition and toward understanding the biological basis of this phenomena in other early-onset genetic forms of PD.

Here, we review the clinical evidence for early hyperkinetic symptoms in patients with *PARKIN* mutations and the recent evidence that parkin modulates glutamatergic neurotransmission. We discuss the hypothesis that early hyperkinetic symptoms of PD patients with *PARKIN* mutation may be considered manifestations of corticostriatal synaptopathy. These features can be linked to dopamine depletion, a well-recognized mechanism in the literature, but they also may be related to a potential direct effect of parkin on the corticostriatal synapse.

## Clinical Evidence for Dystonia and Dyskinesia in Parkin Patients

Since the first description of juvenile parkinsonism caused by mutations of the *PARKIN* gene in two families (Kitada et al., 1998), dystonia has been proposed as a clinical hallmark of the disease (Schrag and Schott, 2006). Recent literature reviews have reported that an overall, clinically typical form of PD with early onset, slow progression, and excellent response to levodopa treatment is frequently associated with dystonia and dyskinesia in patients with *PARKIN* mutations (Grünewald et al., 2013; Kasten et al., 2018). Usually independent of levodopa intake, dystonia is described as the presenting symptom in a large percentage of *PARKIN* patients in whom it can be present in isolation for years before the appearance of parkinsonism (Khan et al., 2003). The reported phenotypic overlap between PD patients with *PARKIN* mutation and rare forms of dystonia-parkinsonism, such as levodopa-responsive dystonia (DRD), prompts screening for *PARKIN* mutations along with *GTP cyclohydrolase* 1 and *tyrosine hydroxylase* in clinically diagnosed DRD patients (Tassin et al., 2000; Wu et al., 2005, 2008; Schneider and Bhatia, 2010; Potulska-Chromik et al., 2017). To determine whether dystonia contributes to gait abnormalities in patients with *PARKIN* mutations, a recent study used a clinical computerized video motion analysis system to evaluate lower limb dystonia severity in 15 patients. Lower limb dystonia occurred in most *PARKIN* patients who displayed a specific, abnormal gait pattern that differed from the patterns observed in healthy controls independent of the OFF/ON state (Castagna et al., 2016).

In addition to dystonia, *PARKIN* patients show dyskinesia at exceedingly low dosages of levodopa as compared with late-onset PD patients. On average, the response to low doses of levodopa is reported as being excellent and sustained. However, the likelihood of developing LID is reportedly higher than in individuals with parkinsonism resulting from other aetiologies (Brüggemann and Klein, 1993; Paviour et al., 2004) and it is inducible by very-low-dose levodopa in some cases (Khan et al., 2003). While bradykinesia responds well even to low levodopa dosages in *PARKIN* patients, the same low doses seem to exacerbate dystonia already in very early stages of the disease. Although conflicting results have also been reported (Kasten et al., 2018), the present evidence suggests that the incidence of hyperkinetic disorders at earlier disease stages is higher in *PARKIN* patients than in those with iPD.

## Potential Molecular Mechanisms Underlying Dystonia and Dyskinesia

**FIGURE 1:**
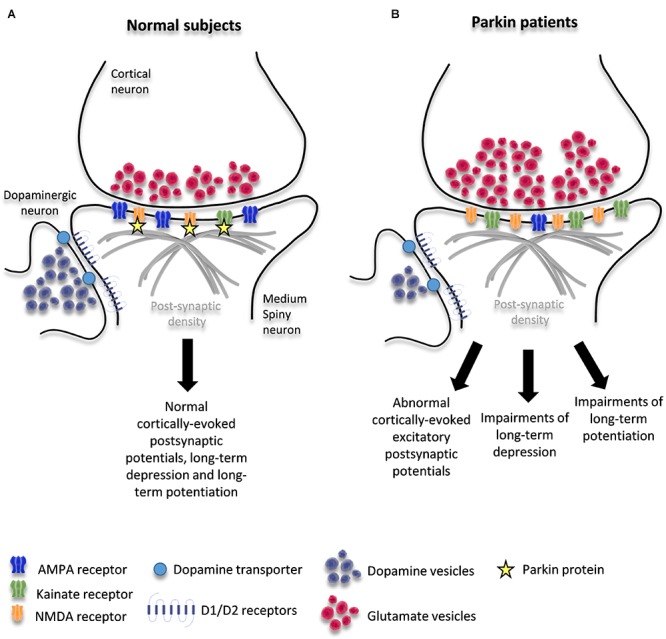
Schematic diagram of corticostriatal synapse in healthy condition **(A)** and in patients with *PARKIN* mutations **(B)**. The figure shows the interaction of parkin with KAR and NMDAR and potential changes induced by loss of *PARKIN* function.

The precise mechanisms underlying dystonia and LID in PD are unknown. Studies on PD animal models, mainly dopamine-depleted animals, and PD patients strongly suggest that dyskinesia results from dysfunction of glutamatergic transmission from the cortex to the striatal medium spiny neurons (MSNs) (Picconi et al., 2017). MSNs are characterized by radially projecting dendrites densely studded with spines and receiving excitatory glutamatergic inputs from the cerebral cortex which form in the order of 5000 contacts per neuron (Graveland et al., 1985; David Smith and Paul Bolam, 1990; Yelnik et al., 1991). The dendritic spines bear the asymmetrical glutamatergic synapse on the head and the symmetric dopaminergic synapses on the neck (Figure 1A; David Smith and Paul Bolam, 1990). This characteristic synaptic triad boosted the widely accepted theory that dopamine (DA) physiologically modulates the strength of cortical inputs to the MSNs (Gerfen and Surmeier, 2011) and that long-term DA depletion occurring over the course of idiopathic PD triggers prominent secondary changes in the corticostriatal synapses (Picconi et al., 2017). Indeed, DA depletion leads to strong sensitization of post-synaptic D1 receptors, which is critical for the development of dyskinesia (Darmopil et al., 2009), and induces a variety of molecular changes in the downstream signaling pathways, including an increase in the state of phosphorylation of the DA- and cAMP-regulated phosphoprotein of 32 KDa (DARPP-32) (Santini et al., 2007; Heumann et al., 2014; Ruiz-Dediego et al., 2015; Solís et al., 2017). DARPP-32 phosphorylation promotes a cascade of intracellular events in MSNs: activation of extracellular signal-regulated kinase 1 and 2 (ERK1 and 2) and, as a consequence, activation of the ERK and the mammalian target of rapamycin complex 1 (mTORC1) signaling pathways (Santini et al., 2012; Heumann et al., 2014).

The molecular signatures that have recently emerged as key signals in LIDs are ERK1/2 phosphorylation (Pavón et al., 2006), abnormal activation of mitogen- and stress-activated protein kinase-1 (MSK-1), and phosphorylation of histone H3 (Santini et al., 2007; Heumann et al., 2014). The final result of this chain of events is a change in the molecular make-up of MSNs due to alterations in gene expression and protein synthesis that might account for maladaptive morphological plasticity at the corticostriatal synapse (Picconi et al., 2017). Morphological examination of MSNs in dopamine-depleted animals and in autopsy specimens from iPD patients has revealed dendrite atrophy, truncated dendrites, and decreased spine density (Stephens et al., 2005; Zaja-Milatovic et al., 2005; Suarez et al., 2016). These functional and morphological changes in the corticostriatal synapses are believed to result in dyskinesias (Picconi et al., 2017).

The question arises as to whether the same molecular mechanisms underlie the early and atypical dyskinesia displayed by *PARKIN* patients. Clinical studies with [18F] Dopa as a marker of the functional integrity of pre-synaptic dopamine nerve terminals have shown that *Parkin* mutated patients have widespread pre-synaptic dopaminergic deficits (Scherfler et al., 2004). So while the early hyperkinetic symptoms in patients with parkin mutations can be linked to DA depletion, the appearance of dyskinesia in early disease stages, before the onset of typical and cardinal signs of parkinsonism, suggests that additional mechanisms may be involved. In particular, dyskinesia-related molecular changes in patients with *PARKIN* mutation may partially stem from dysfunctions of the corticostriatal synapses due to the loss of parkin function in these synapses.

## The Potential Role of Parkin at the Corticostriatal Synapse

*In vitro* studies have revealed that parkin can localize at the pre-synapse, where it associates with the cytoplasmic surface of synaptic vesicles (Kubo et al., 2001; Mouatt-Prigent et al., 2004) and binds to synaptotagmin-11, a pre-synaptic protein involved in synaptic vesicle formation, docking, and recycling (Huynh et al., 2003; Wang et al., 2018). The loss of parkin function may inhibit endocytosis and the processes of vesicle replenishment and recycling, leading to as yet undefined changes in neurotransmitter release. Interestingly, the pre-synaptic functions of parkin resemble the function of synuclein, another key protein involved in PD pathogenesis and a regulator of pre-synapse size and synaptic vesicle pool organization (Anwar et al., 2011; Vargas et al., 2017), additionally, the roles of other PD genes such as *DNAJC6*, *SYNJ1*, *SH3GL2, LRRK2*, and *VPS35* in the regulation of synaptic vesicle trafficking SVE are beginning to emerge (Nguyen et al., 2018).

*In vitro* studies on glutamatergic neurons showed that parkin can also localize at the post-synapse, where it directly modulates ionotropic glutamate receptor function. Parkin directly interacts with and ubiquitinates the GluK2 subunit of kainate receptor (KAR) and thus regulates its function: loss of parkin increases KAR levels in the human cortex and KAR function in glutamatergic hippocampal neurons *in vitro* (Maraschi et al., 2014). At the post-synapse, loss of parkin function probably leads to dysregulation of other neurotransmitter receptors as well: a study on brain tissues from parkin knockout mice showed up-regulation of KAR density, increased NMDA receptor density, and reduced AMPA receptor density throughout various brain regions (Cremer et al., 2015). The role of parkin in modulating AMPA receptors was further confirmed in an *in vitro* study showing that parkin-deficient hippocampal neurons exhibit significantly reduced AMPA receptor-mediated currents (Cortese et al., 2016). The suggested underlying mechanism is the decrease in the post-synaptic expression of the adaptor protein Homer1, which is necessary for coupling AMPAR endocytic zones with post-synaptic density. A more recent study evaluated the impact of four parkin point mutations on glutamatergic synaptic function in hippocampal neurons and found that these mutants alter NMDA and AMPA receptor-mediated currents and cell-surface levels and that they prevent the induction of long-term depression. The study demonstrated that parkin regulates NMDA receptor trafficking through ubiquitination of the subunit GluN1 and that the mutations impair ubiquitination activity (Zhu et al., 2018). Hence, the bulk of evidence strongly suggests that *PARKIN* mutations disturb glutamatergic synaptic transmission by directly changing NMDAR, AMPAR, and KAR receptor function. Since parkin is widely expressed in various brain regions and neuron types, including cortical neurons and MSNs, (Kitada et al., 1998; Stichel et al., 2000) it is conceivable that the loss of parkin directly induces pathological effects on these neurons and contributes to the pathophysiology of dyskinesia.

These studies on the localization of parkin and its capacity to directly regulate excitatory transmission constitute an important step toward understanding the functions of parkin at the synapses (Sassone et al., 2017). The complementary approach was to investigate the function of nigrostriatal and corticostriatal synapses in murine models knockout for *Parkin* gene. Unfortunately, studies on the nigrostriatal pathway revealed many discrepancies probably deriving from different experimental conditions and/or from *in vivo* age-dependent compensatory mechanisms. Increased spontaneous DA release from SNc DA neurons was found in *Parkin*-knockout mice aged 8 to 9 months (Goldberg et al., 2003). Decreased evoked DA release was found in 3-to-9-month-old *Parkin* knockout mice (Oyama et al., 2010). In the striatum of 22-month-old wild-type and *Parkin*-knockout mice, DA levels were reported to be indistinguishable (Perez and Palmiter, 2005). DA overflow in striatal slices from 6-to-8-week-old mice was found to be equal between wild-type and *Parkin*-knockout mice (Sanchez et al., 2014). Indeed, not all the evidence supports the idea that DA release is impaired in the nigrostriatal pathway of *Parkin*-knockout mice. Studies on corticostriatal synapses performed by intracellular recordings of MSNs revealed impairment in long-term depression and long-term potentiation in *Parkin*-knockout mice (Kitada et al., 2009). These results suggest that parkin is involved in the regulation of striatal synaptic plasticity. Unfortunately, however, *Parkin*-knockout mice do not show DA neuron loss, motor impairment, dystonic phenotype or hypersensitivity to levodopa (Perez and Palmiter, 2005). The authors suggest that this murine model may represent a tool to study the early stages of the disease (Dawson et al., 2010); nonetheless, its usefulness as a disease model is limited and complicates the interpretation of the results obtained on these mice. More promising results come from studies on dopaminergic neurons differentiated from induced human pluripotent stem cells. When glutamatergic transmission was studied in a mixed population of GABAergic, glutamatergic, and dopaminergic neurons differentiated from induced human pluripotent stem cells, *PARKIN* mutations were observed to potentiate glutamatergic transmission by modifying synaptic vesicle function at the pre-synaptic site of the corticostriatal synapse (Zhong et al., 2017). Moreover, the actions of dopamine on D1-class receptors were noted to be markedly altered in dopaminergic neurons differentiated from induced human pluripotent stem cells of PD patients with *PARKIN* mutations. In particular, as compared with control neurons, the parkin-deficient neurons had a stronger response to the activation of D1-class dopamine receptors (Zhong et al., 2017).

This raises the possibility that parkin could be involved in D1 receptor hypersensitization after dopaminergic denervation, an important mechanism underlying the dyskinesia in iPD (Heumann et al., 2014). Accordingly, a recent paper showed that parkin ubiquitinates and regulates the levels of STEP61, the striatal enriched protein tyrosine phosphatase, whereas clinically relevant parkin mutants fail to do so. Because STEP61 substrates include ERK1/2, it is conceivable that a parkin-induced increase in STEP61 might influence D1 signaling in MSNs (Kurup et al., 2015).

Taken together, these findings suggest that parkin orchestrates a complex remodeling of corticostriatal synapses through multiple mechanisms. These mechanisms are likely to include changes due to DA depletion as well as those due to loss of parkin function at these synapses: alterations in the trafficking of glutamate vesicles at the glutamatergic pre-synapse and/or dysregulation of glutamate receptor levels on MSNs and/or dysregulation of D1-signaling. These mechanisms may be responsible for the precocious maladaptive plasticity of the corticostriatal synapses that leads to early dyskinesia (Figure 1B).

## Conclusion

Since the parkin protein modulates glutamatergic receptor function and possibly glutamate release as well, it is conceivable that *PARKIN* mutation affects corticostriatal transmission, leading to early maladaptive changes in this glutamatergic synapse. This alteration could produce changes due to corticostriatal-induced depletion of DA at the synapses. What does this scenario imply for the development of specific and effective treatments? The only currently available drug for symptomatic treatment of dyskinesia is amantadine, an antiviral agent introduced in the early 1970s that also displays NMDA receptor antagonism (Hubsher et al., 2012). With a better understanding of the mechanisms by which parkin causes early dystonia and dyskinesia, researchers may be able to identify novel therapeutic targets and develop strategies that include drugs that act on the glutamatergic system. Such drugs are already under investigation for treating dyskinesias in iPD (Müller, 2017). Moreover, if our hypothesis proves true, the distinct molecular mechanisms underlying dyskinesia between *PARKIN* patients and late-onset PD patients could be relevant for devising precision medicine strategies for parkin patients. The recent discovery that LID is caused by a distinct and stable group of striatal neurons (Girasole et al., 2018) paves the way to further studies aimed at investigating the role of parkin in these neurons and at their corticostriatal synapse.

In conclusion, published data support the hypothesis of an early corticostriatal synaptopathy in parkin patients caused by parkin modulating the glutamatergic synapse. This hypothesis could provide a biological basis for studying the disease phenotype and lay the groundwork for the pharmacological treatment of PD patients with *PARKIN* mutation.

## Author Contributions

JS wrote first draft of the manuscript. FV and AC reviewed and critically revised the manuscript.

## Conflict of Interest Statement

The authors declare that the research was conducted in the absence of any commercial or financial relationships that could be construed as a potential conflict of interest.
